# (2 + 1)D‐CAIPIRINHA accelerated MR spectroscopic imaging of the brain at 7T

**DOI:** 10.1002/mrm.26386

**Published:** 2016-08-22

**Authors:** B. Strasser, M. Považan, G. Hangel, L. Hingerl, M. Chmelik, S. Gruber, S. Trattnig, W. Bogner

**Affiliations:** ^1^ MRCE, Department of Biomedical Imaging and Image‐Guided Therapy, Medical University of Vienna Vienna Austria; ^2^ Christian Doppler Laboratory for Clinical Molecular MR Imaging, Medical University of Vienna Vienna Austria

**Keywords:** CAIPIRINHA, parallel imaging, magnetic resonance spectroscopic imaging, brain MRSI, CSI, 7 Tesla

## Abstract

**Purpose:**

To compare a new parallel imaging (PI) method for multislice proton magnetic resonance spectroscopic imaging (^1^H‐MRSI), termed (2 + 1)D‐CAIPIRINHA, with two standard PI methods: 2D‐GRAPPA and 2D‐CAIPIRINHA at 7 Tesla (T).

**Methods:**

(2 + 1)D‐CAIPIRINHA is a combination of 2D‐CAIPIRINHA and slice‐CAIPIRINHA. Eight healthy volunteers were measured on a 7T MR scanner using a 32‐channel head coil. The best undersampling patterns were estimated for all three PI methods. The artifact powers, g‐factors, Cramér–Rao lower bounds (CRLB), and root mean square errors (RMSE) were compared quantitatively among the three PI methods. Metabolic maps and spectra were compared qualitatively.

**Results:**

(2 + 1)D‐CAIPIRINHA allows acceleration in three spatial dimensions in contrast to 2D‐GRAPPA and 2D‐CAIPIRINHA. Thus, this sequence significantly decreased the RMSE of the metabolic maps by 12.1 and 6.9%, on average, for 4 < R < 11, compared with 2D‐GRAPPA and 2D‐CAIPIRINHA, respectively. The artifact power was 22.6 and 8.4% lower, and the CRLB were 3.4 and 0.6% lower, respectively.

**Conclusion:**

(2 + 1)‐CAIPIRINHA can be implemented for multislice MRSI in the brain, enabling higher accelerations than possible with two‐dimensional (2D) parallel imaging methods. An eight‐fold acceleration was still feasible in vivo with negligible PI artifacts with lipid decontamination, thus decreasing the measurement time from 120 to 15 min for a 64 × 64 × 4 matrix. Magn Reson Med 78:429–440, 2017. © 2016 The Authors Magnetic Resonance in Medicine published by Wiley Periodicals, Inc. on behalf of International Society for Magnetic Resonance in Medicine.

## INTRODUCTION

Proton magnetic resonance spectroscopic imaging (^1^H‐MRSI) can aid in the diagnosis of several brain diseases, such as tumors 1, 2, multiple sclerosis 3, 4, or mild traumatic brain injuries 5. Yet, the long measurement times and low signal‐to‐noise ratio per unit time (SNR/t) in vivo prevent its widespread acceptance in the clinical routine.

The SNR/t can be improved in several ways, among which are the use of high magnetic fields 6, array coils (AC) 7, an optimal coil combination that includes noise decorrelation 8, short echo times (TE) 6, efficient sequences, and short repetition times (TR) 9. The excess SNR can then be traded off for a reduced acquisition time through i) measuring several k‐space points within each spectral dwell time (ie, spatio‐spectral encoding (SSE)) 9; ii) reducing the number of sampled k‐space points (ie, partial Fourier, compressed sensing, parallel imaging (PI)) 10; or iii) reducing the TR (ie, steady‐state free precession sequences) 11.

SSE in MR spectroscopic imaging (MRSI) was proposed with several different gradient trajectories, eg, spiral 12, echo‐planar spectroscopic imaging 13, 14, proton echo‐planar spectroscopic imaging 15, 16, rosette 17, and CONCEPT 18. SSE spectroscopy techniques often have high‐gradient hardware requirements 19. Otherwise, SSE sequences are very well suited for accelerating MRSI, but even more promising is the combination of SSE techniques with PI or compressed sensing 20.

PI techniques were shown to be versatile tools for accelerating phase‐encoding 21, 22, 23, as well as slice encoding in multislice sequences 24. For GRAPPA and SENSE, whole k‐space lines are omitted, whereas, for 2D‐CAIPIRINHA, k‐space points are skipped in arbitrary patterns. The missing data are then reconstructed with the aid of the intrinsic signal localization of the individual AC elements, ie, different sensitivity profiles for different AC elements, and additional calibration data. This reconstruction can be performed either in k‐space (GRAPPA) or in the image domain (SENSE). In slice‐CAIPIRINHA, several slices are excited at once with a variable field of view (FOV) shift between the slices; thus, this is also termed simultaneous multislice (SMS) acquisition 25. The individual slices are then aliased on top of each other in the measured data and have to be unfolded using concepts similar to those used in phase‐encoding PI.

Special care must be taken with subcutaneous lipids when applying PI in brain MRSI. If subcutaneous lipids cannot be fully unaliased, this signal can contaminate voxels within the brain and can even superimpose the main metabolite resonance in the brain, N‐acetyl‐aspartate (NAA), when strong B_0_ inhomogeneities are present 26. Apart from this, PI in MRSI has many advantages. PI in MRI is a versatile tool, yet, in ^1^H‐MRSI, the efficiency is even higher, because the ratio between the actual scan time and the calibration scan time is better than in conventional MRI. In addition, PI in n‐dimensional, phase‐encoded MRSI can be performed along more spatial dimensions than in n‐dimensional, Cartesian MRI, as conventional MRSI has no frequency‐encoding direction. The performance of PI was shown to improve with the magnetic field strength 27, whereas some ultra‐high‐resolution SSE MRSI sequences are slightly less efficient at very high magnetic fields 9. However, the efficiency loss is negligible for voxel sizes ≥ 200/32 mm, or at field strengths ≤ 3 T.

Several groups have implemented different versions of PI in MRSI, such as one‐dimensional (1D) SENSE in combination with SSE 28, 29, 30, 31, 32, 33, two‐dimensional (2D) SENSE 34, 35, 36, 37, 38, 39, 1D‐GRAPPA in combination with SSE 14, 29, 40, 41, and 2D‐GRAPPA 42. To our knowledge, no group has presented any slice‐PI method in MRSI as of yet.

PI is available along potentially three spatial dimensions in MRSI. The performance of PI was shown to increase with the number of spatial dimensions 43. Therefore, we propose (2 + 1)D‐CAIPIRINHA as a combination of 2D‐CAIPIRINHA along the two axial phase‐encoding directions, and slice‐CAIPIRINHA along the slice‐encoded third dimension, to accelerate multislice ^1^H‐MRSI acquisitions. A similar method can be used for three‐dimensional (3D) ^1^H‐MRSI.

## METHODS

### Description of (2 + 1)D‐CAIPIRINHA

**Figure 1:**
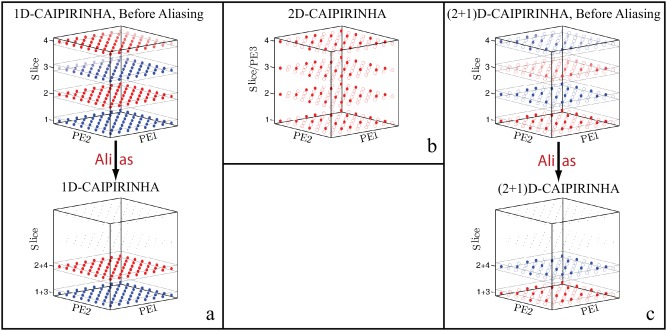
Illustration of three different methods by which to accelerate MRSI data. Each sphere represents a k‐space point in an 8 × 8 × 4 k‐space, while the gray boxes represent slices. The different colors represent different slices. (a) 1D‐CAIPIRINHA: Four different slices are excited in 1D‐CAIPIRINHA, and Slices 1 and 3, and Slices 2 and 4 are aliased on top of each other during measurement, as depicted in the bottom. The transparency of the spheres represents the linear phase shift, causing an FOV shift between the aliased slices. (b) 2D‐CAIPIRINHA: Four slices are measured with 2D‐CAIPIRINHA. Each slice/partition is independently measured and undersampled with the same 2D pattern. (c) (2 + 1)D‐CAIPIRINHA: The combination of slice‐CAIPIRINHA and 2D‐CAIPIRINHA leads to (2 + 1)D‐CAIPIRINHA, in which Slices 1 and 3, and Slices 2 and 4 are aliased on top of each other with an FOV shift between them; each slice is also undersampled with a 2D‐CAIPIRINHA pattern.

(2 + 1)D‐CAIPIRINHA is a combination of 2D‐CAIPIRINHA 23 and the original “slice” CAIPIRINHA 24, both by Breuer et al. In slice‐CAIPIRINHA, a number of different slices are excited at once (Fig. 1a, top). These slices are aliased and can be shifted in‐plane with respect to each other to enhance the reconstruction quality (Fig. 1a, bottom). The in‐plane shift is indicated by the transparency of the k‐space points, showing the linear phase along the phase‐encoding axes. Figure 1b depicts 2D‐CAIPIRINHA. The combination of both methods is shown in Figure 1c, resulting in (2 + 1)D‐CAIPIRINHA. This combination offers an increased encoding freedom, namely, the 2D‐CAIPIRINHA pattern, and the two FOV shifts in both phase‐encoding directions.

In contrast, 3D‐CAIPIRINHA would be an extension of 2D‐CAIPIRINHA to three phase‐encoded spatial dimensions. As far as we know, 3D‐CAIPIRINHA has not yet been proposed, but similar concepts were proposed by Bilgic et al 44 and Breuer et al 45 for MRI.

In both cases, (2 + 1)D‐ and 3D‐CAIPIRINHA, the sensitivity variations of the AC are exploited in all three spatial dimensions.

### Subjects and Hardware

Eight healthy volunteers were measured on a 7 Tesla (T) whole‐body MR scanner (Magnetom, Siemens Healthcare, Erlangen, Germany) with a 32‐channel AC for signal reception, and a volume coil for signal transmission (Nova Medical, Wilmington, Massachusetts, U.S.A.). Two measurements had to be excluded as a result of motion artifacts caused by the long measurement time (see subsequently). The remaining six data sets were further processed. The local institutional review board approved this study, and written, informed consent was obtained from all volunteers.

### Data Acquisition

As an anatomical reference, a 3D T_1_‐weighted magnetization‐prepared 2 rapid acquisition gradient echoes (MP2RAGE) sequence was acquired 46. The sequence parameters were as follows: TE = 2.96 ms; TR = 4.2 s; inversion time 1 = 0.85 s; inversion time 2 = 3.4 s; GRAPPA factor 3; matrix size 256 × 256 × 160; and nominal voxel size 0.9 × 0.9 × 1.1 mm³. A $B_{1}^{+}$ map was acquired with a presaturation turboFLASH‐based B_1_ mapping sequence 47, 48 to adjust the intended average flip angle of 45 ° in the MRSI sequence. The shim volume was carefully placed to cover the whole head in the transverse planes, including the subcutaneous fat around the brain, to avoid remaining lipids from unshimmed regions superimposing NAA. To check the shimming quality, a B_0_ field map was acquired with a double gradient echo sequence.

To enable a retrospective comparison of different PI approaches in simulations, elliptically weighted, fully sampled MRSI data had to be acquired without any acceleration, which could then be used as the reference standard. The different acceleration methods were simulated in postprocessing. Due to time restrictions, only two slices were fully sampled to show the feasibility of slice acceleration. The two MRSI slices were pulse‐cascaded, Hadamard‐encoded 49, 50, and acquired with an FID‐based sequence 51. The pulse‐cascaded Hadamard scheme is similar to normal Hadamard encoding, in which all slices are excited with pulse phases according to a Hadamard matrix. In contrast to conventional Hadamard encoding, the pulses for the slices are not summed, but instead are transmitted one after another. The parameters of the sequence were 64 × 64 × 2 voxels; elliptically weighted; nominal voxel size 3.4 × 3.4 × 8 mm³; slice gap of 8 mm; 6‐kHz spectral bandwidth; 2048 complex free induction decay (FID) points; weak water suppression enhanced through T1‐effects (WET) 52; TR of 0.6 s; flip angle of 45 °; acquisition delay of 1.3 ms (upper slice) and 2.3 ms (bottom slice); and measurement time of 60 min. Two slices were acquired with a gradient echo sequence to serve as GRAPPA auto‐calibration signals (ACS) and as coil combination weights 8, and were measured with the same FOV, slice positions, shim adjustments, and echo times (same as acquisition delays), but without water suppression and with a 128 × 128 matrix size.

In addition, undersampled MRSI and gradient‐echo MRI data with four slices were acquired in one volunteer (Volunteer #6) with an acceleration of R_Total_ = 8, R_Slice_ = 2, and Hadamard encoding of the resulting two slice groups, to show the feasibility of the proposed method for more slices. The slices were measured without gaps.

### Quality Measures

Two important quality measures of PI are the artifact power (AP) and the g‐factor. The artifact power is defined as follows:

(1) $$AP:=100\cdot \frac{\sum_{cha}\sum_{\vec{r}\in mask}\sum_{t\in Tmask}\;\;\left|S_{Accel}\left(cha,\vec{r},t\right)-S_{Full}\left(cha,\vec{r},t\right)\right|}{\sum_{cha}\;\sum_{\vec{r}\in mask}\;\sum_{t\in Tmask}\;\;\left|S_{Full}\left(cha,\vec{r},t\right)\right|}$$

where cha are the different channels, *r* is the voxel position, *t* is the FID time points, *mask* is the spatial brain mask, *Tmask* is the mask in the time domain (that takes every eighth point from the first 512 FID points to reduce computational burden), and *S* is the signal of either the simulated acceleration or of the full data set.

Therefore, the AP is a measure of the relative error of the time domain signal in percent.

Acceleration through the omission of k‐space points leads to an expected loss in SNR as a result of fewer independent measurements. The g‐factor describes the additional SNR loss that is caused by imperfect reconstruction. The g‐factor is defined as

(2) $$g:=\frac{SNR_{Full}}{\sqrt{R_{Total}}SNR_{PI}}$$

Writing the SNR as the ratio of the signal and the root mean square (RMS) of the noise, and assuming that the signal is unchanged between the PI and the normal acquisition, Equation [2] can be rewritten as

(3) $$g≔\frac{Signal_{Full}}{RMS\left(Noise_{Full}\right)}/\frac{\sqrt{R_{Total}}Signal_{PI}}{RMS\left(Noise_{PI}\right)}\approx \frac{RMS\left(Noise_{PI}\right)}{\sqrt{R_{Total}}\cdot RMS\left(Noise_{Full}\right)}$$

This formula was used throughout the manuscript for calculating the g‐factors. R_Total_ was defined as the total acceleration factor with respect to the elliptically sampled, full data, taking into account the in‐plane acceleration, R_InPlane_, the slice acceleration, R_Slc_, and the variable density (VD) radius, ie, the radius within which all k‐space points were “measured.”

Another quality measure is the RMSE of the metabolic maps, as follows:

(4) $$RMSE:=100\cdot \sqrt{\frac{\sum_{r\in mask}{\left(\frac{C_{Accel}\left(\vec{r}\right)-C_{Full}\left(\vec{r}\right)}{C_{Full}\left(\vec{r}\right)}\right)}^{2}}{N}}$$

where *C* is the fitted concentration of one specific metabolite, and *N* is the number of voxels in the mask.

### Identifying the Best Undersampling Patterns

Our first goal was to identify the best (2 + 1)D‐CAIPIRINHA, 2D‐CAIPIRINHA, and 2D‐GRAPPA patterns among all possible patterns. The (2 + 1)D‐CAIPIRINHA patterns consisted of a 2D‐CAIPIRINHA pattern and two FOV shifts in both phase‐encoding directions between the two slices.

The starting point for the 2D‐CAIPIRINHA patterns was all possible patterns arising when distributing *j* points in a k × k undersampling cell with the intended in‐plane acceleration, fulfilling $R_{InPlane}\approx j/k^{2}$. This was calculated for all possible *j* and *k*, restricted only by requiring the number of resulting patterns to be below 10^8^. The GRAPPA patterns were calculated by choosing natural numbers *n* and *m*, such that $R_{InPlane}\approx n\cdot m$. The best (2 + 1)D‐CAIPIRINHA patterns were estimated in three steps.

#### Step 1: k‐Space Distance

Suitable 2D‐CAIPIRINHA patterns were identified by minimizing the mean distance between a nonmeasured k‐space point and its three measured next neighbors. Such patterns tend to result in better reconstruction performance, as nearby k‐space points contribute the most to the reconstruction information 21. The 20 patterns with the lowest mean distance were chosen for Step 2.

#### Step 2: Artifact Power and g‐Factor Minimization

Step 2 was performed for four volunteers. The volunteer mean was computed at the end. Each of the substeps, 2a and 2b, resulted in one pattern, minimizing either the artifact power or the g‐factor. Both were used for Step 3. In some cases, the patterns of 2a and 2b coincided, in which case only one pattern was processed in Step 3.

a. **Artifact Power**: The combinations of those 2D‐CAIPIRINHA patterns and FOV shifts $\in {\left\{0,\frac{1}{6},\frac{2}{6},\frac{3}{6}\right\}}_{x}\cdot {\text{FoV}}_{x}\times {\left\{0,\frac{1}{6},\frac{2}{6},\frac{3}{6}\right\}}_{y}\cdot {\text{FoV}}_{y}$ were selected, which minimized the artifact power within the brain mask, as defined by Equation [1]. The FOV shifts were simulated in both phase‐encoding directions independently. The brain mask was defined based on the T_1_‐weighted image of the MP2RAGE data set, using the brain extraction tool BET2 (http://www.fmrib.ox.ac.uk/analysis/research/bet).
b. **g‐Factors**: For each of the 20 best patterns in Step 1, the 0.9‐quantile of the g‐factors within the brain mask was calculated, independent of Step 2a. The pattern with the minimum g‐factor was chosen for Step 3, along with the pattern of 2a. This measure was chosen because the highest g‐factors restrict the maximum possible acceleration, but the maximum g‐factor is prone to outliers. The g‐factors were computed as the ratio of the RMS of the undersampled and the fully sampled noise (Eq. [3]).

#### Step 3: Root Mean Square Error

Step 3 was performed for five volunteers. The volunteer mean was computed at the end of Step 3.

Finally, the data were undersampled independently with the best patterns of 2a and 2b, and then reconstructed and processed using LCModel. The RMSE of total N‐acetyl‐aspartate (tNAA) was used to determine the overall best pattern of the given R_Total_. tNAA was chosen, as residual lipid contamination from non–fully unaliased lipids occur primarily in the tNAA signal. The details about computing the RMSE are described subsequently.

The whole procedure was performed for different R_Total_ ∈{2,3,…,10} (2D‐GRAPPA, 2D‐CAIPIRINHA) or R_Total_ ∈{  5,6,…,10 } ((2 + 1)D‐CAIPIRINHA).

The best 2D‐CAIPIRINHA patterns were estimated similarly, but without the slice aliasing and reconstruction part. As a result, higher R_InPlane_s were necessary to achieve the same R_Total_. To obtain the best 2D‐GRAPPA patterns, the same procedure was performed with simple n × m GRAPPA patterns, requiring $R_{Total}=n\cdot m$ (n,m integer and n,m < 6). If this resulted in high‐acceleration factors along one spatial dimension, eg, for R_InPlane_ = 5, the next higher possible R_InPlane_ was chosen and compensated by a higher VD radius. Both possibilities, n × m and m × n, were simulated. Thus, eg, R_Total_ = 5 was achieved by four patterns in 2D‐GRAPPA: 1 × 5; 5 × 1; 2 × 3 with VD radius = 6; and 3 × 2 with VD radius = 6. By allowing more patterns, the quality measures, especially of GRAPPA, can be dramatically improved for R_Total_ = 5, 7, 8, and 10.

As a mean of flexibility, the VD radius could also be chosen to adapt to slightly different R_Total_s for 2D‐CAIPIRINHA and (2 + 1)D‐CAIPIRINHA. The RMSE was calculated as follows: The noise‐decorrelated full data set was undersampled according to the used pattern, and reconstructed by first performing a k‐space‐based in‐plane reconstruction algorithm, and then, for the (2 + 1)D‐CAIPIRINHA, a k‐space‐based slice reconstruction algorithm. The data were then spatially Fourier transformed, coil‐combined using MUSICAL 8, and Hamming‐filtered. The individual spectra were fitted with LCModel (http://s-provencher.com/pages/lcmodel.shtml) using a basis set with metabolites simulated by “NMR scope” of jMRUI (http://www.mrui.uab.es). These simulated metabolites contain the same acquisition delay as the MRSI data 51; thus, the first‐order phase error is accounted for. For the presentation in this publication, the spectra were first‐order phase‐corrected with a MATLAB (MathWorks, Natick, Massachusetts, USA) routine, and the resulting increased baseline variations were fitted by LCModel as the baseline. The RMSE of the tNAA map was calculated with the fully sampled data set as the gold standard.

### Analyzing the Best Undersampling Patterns

The best patterns of Step 3 (minimum RMSE) were compared among the three PI methods for the volunteer mean and all R_Total_ values using RMSEs and Cramér–Rao lower bound (CRLB) values as a measure of PI reconstruction quality. Statistical tests were performed on the difference between (2 + 1)D‐CAIPIRINHA and the two other methods, using the APs, g‐factors, RMSE, and CRLB. Spectra and metabolic maps were compared qualitatively among the three PI methods.

### Lipid Contamination and Feasibility of (2 + 1)D‐CAIPIRINHA

To determine the sensitivity of all three PI methods to lipid artifacts, the lipid content relative to the reference with R_Total_ = 1 was evaluated by summing the magnitude spectra in the range of 0.3 to 2.1 ppm, dividing by the results of the reference, and averaging over the brain voxels and the volunteers. This was done for different values of R_Total_, and undersampling methods. By choosing such a broad range in parts per million, all lipids, even those overlapping with tNAA, are quantified, with the drawback of having “contamination” values > 0% even for perfect lipid suppression. In addition, a lipid decontamination algorithm using a regularized reconstruction according to Bilgic et al 53 was performed, and the lipid contamination was again compared for all data sets. In both cases, with and without lipid decontamination, the reference was chosen without lipid decontamination to investigate the lipid suppression capabilities of the regularized reconstruction.

The data from Volunteer #6 were processed with the standard processing described previously, including lipid decontamination, to prove the feasibility of (2 + 1)D‐CAIPIRINHA.

## RESULTS

### Identifying the Best Undersampling Patterns

**Figure 2:**
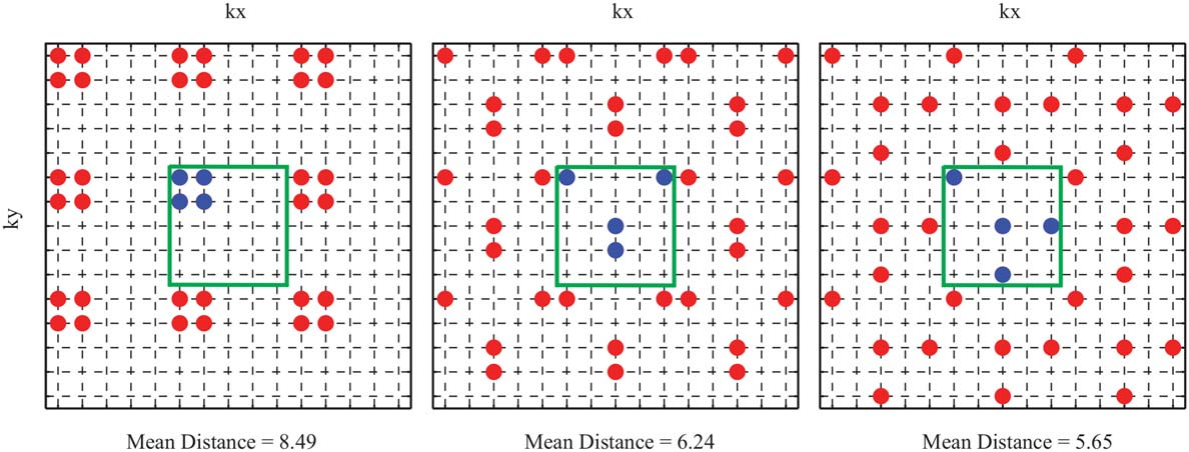
Three examples of the undersampling patterns for R_InPlane_ = 6.25, which are processed in Step 1. The blue points with the green frame represent the elementary cell, which is replicated to the matrix size, in this case, 15 × 15. The mean distance stated below each pattern indicates the mean distance of the nonsampled points to their three measured next neighbors. This measure is used in Step 1 to find evenly distributed patterns.

Three examples of the undersampling patterns of Step 1 (R_InPlane_ = 6.25) are illustrated in Figure 2, together with the mean distance of the nonmeasured k‐space points to their three measured nearest neighbors. It is clearly visible that the mean distance is a good measure of how evenly the measured k‐space points are distributed. An overview of how many patterns were processed in each of the three steps is given in Table 1 for three R_Total_ values.

**Table 1:** Number of Processed Patterns for Each Processing Step and for Three Sample Acceleration Factors

		Step 1	Step 2	Step 3^a^
	2D‐GRAPPA	2	2	2
R = 2	2D‐CAIPI	6438	6	2
	(2 + 1)D‐CAIPI	0	0	0
	2D‐GRAPPA	2	2	2
R = 5	2D‐CAIPI	10.7 · 10^4^	17	2
	(2 + 1)D‐CAIPI	1.3 · 10^6^	576	2
	2D‐GRAPPA	1	1	2
R = 9	2D‐CAIPI	68 · 10^6^	54	2
	(2 + 1)D‐CAIPI	6.7 · 10^6^	400	2

a In step 3, exactly two patterns (or none for (2 + 1)D‐CAIPIRINHA and R < 5) were processed: the best AP pattern (Step 2a) and the best g‐factor pattern (Step 2b).

**Figure 3:**
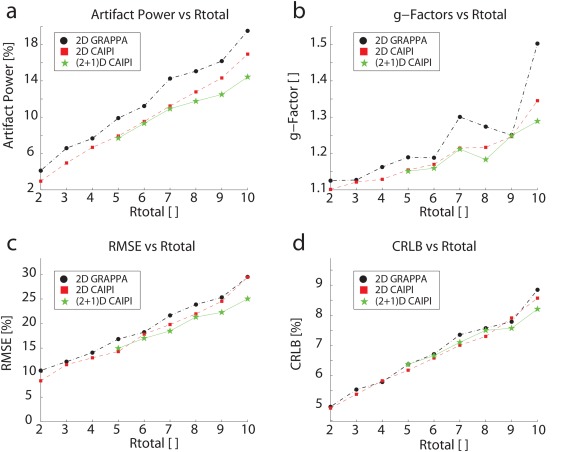
AP (a) and 0.9‐quantile g‐factors (b), averaged over four volunteers, the RMSE of tNAA, averaged over five volunteers (c), and the mean CRLBs of tCho and tCr plotted against the total acceleration, R_Total_ (d). The AP perfectly follows the expected trend: The APs increase with R_Total_ for all PI methods, with (2 + 1)D‐CAIPIRINHA having the smallest, and 2D‐GRAPPA the highest, values. The g‐factors and the RMSE are similar, except for R_Total_ = 9, in which all methods have effectively the same g‐factors. The CRLBs show smaller values only for R_Total_ ≥ 9.

The results of Step 2, ie, the AP and the g‐factors dependent on the R_Total_, are shown in Figures 3a and 3b. Only the results of the best pattern for each PI method are shown. It is clearly visible that the best (2 + 1)D‐CAIPIRINHA pattern achieved smaller APs than the best 2D‐GRAPPA or 2D‐CAIPIRINHA patterns, particularly for high acceleration factors. The APs of (2 + 1)D‐CAIPIRINHA were significantly lower than for 2D‐GRAPPA (see Supporting Table S1). The g‐factors also followed this trend. However, all three methods had very similar g‐factors for R_Total_ = 9.

### Analyzing the Best Undersampling Patterns

The RMSE of the tNAA map for the best undersampling patterns that are dependent on R_Total_ are shown in Figure 3c. The trend is very similar to the results of the AP, with (2 + 1)D‐CAIPIRINHA giving the lowest % RMSE for all acceleration factors higher than R = 5.

**Figure 4:**
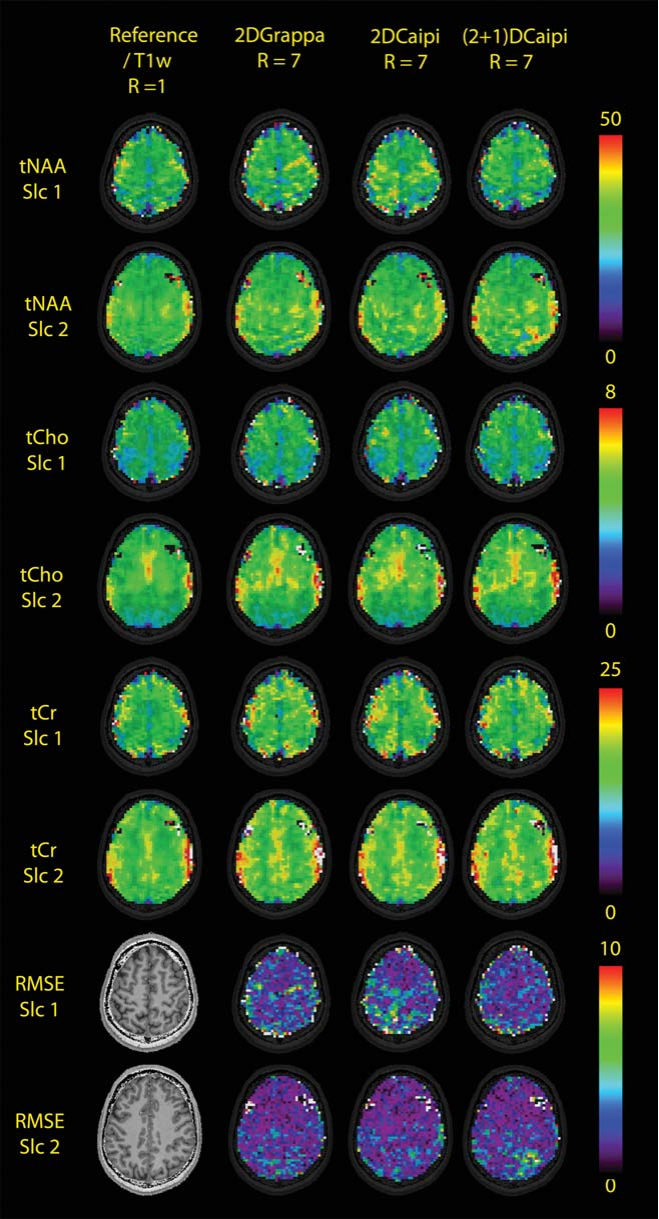
Metabolic maps in native resolution of tNAA, tCho, tCr, and the RMSE values to the R = 1 gold standard for one volunteer with R_Total_ = 7. (2 + 1)D‐CAIPIRINHA shows the least artifacts compared with the fully sampled data for Slice 1, whereas the other two methods perform better in Slice 2. The holes in the upper right and left corner of the tNAA and tCho of Slice 2 are caused by lipid artifacts, which can be removed by the regularized lipid decontaminated reconstruction.

**Figure 5:**
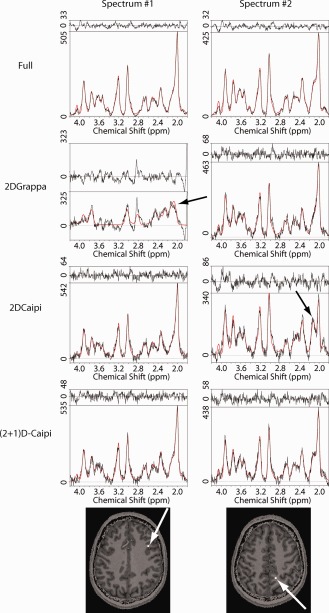
Example of spectra comparing the performance of the three PI methods to the fully sampled data. The first column shows a case in which the 2D‐GRAPPA failed, whereas the other two PI methods performed well. The spectra of the second column demonstrate where the 2D‐CAIPIRINHA is highly lipid contaminated. The black lines in the plots represent the measured data, the red lines the fitted data, and, in the upper part, the residua are plotted. The black arrows in the spectra indicate artifacts caused by remaining lipids. The origin of the spectra are shown on T_1_‐weighted reference images and marked with arrows and white squares in the size of the nominal resolution.

**Figure 6:**
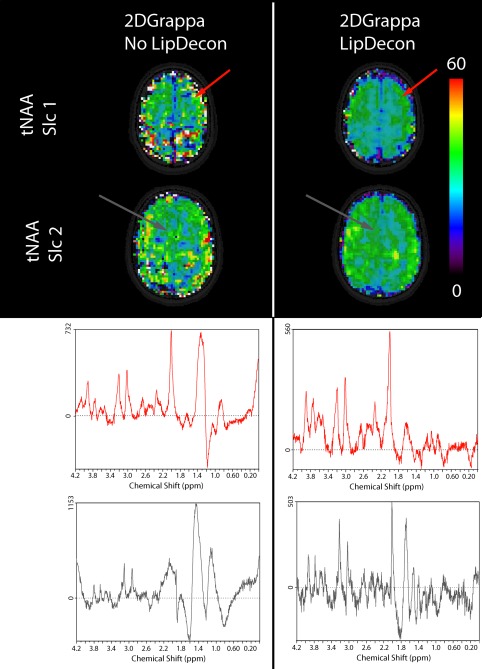
Effect of the lipid decontamination method used to demonstrate one very extreme case of lipid artifacts in Volunteer #5 for 2D‐GRAPPA, R_Total_ = 10. When using the decontamination algorithm, only minor lipid artifacts remain. This fact is additionally shown by the sample spectra from the locations indicated by the red and gray arrows, as the lipid‐decontaminated spectra show much lower lipid signals.

**Figure 7:**
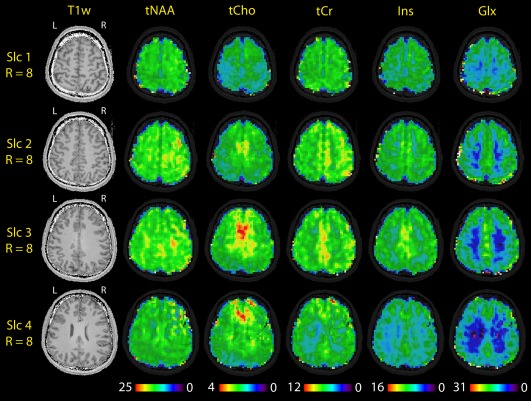
T_1_‐weighted reference and metabolic maps of the additionally measured Volunteer #6, which was undersampled during measurement with (2 + 1)D‐CAIPIRINHA, R_Total_ = 8, leading to a measurement time of 15 min. The metabolic maps tNAA, tCho, tCr, Ins, and Glx are ordered to increase in gray matter/white matter contrast from left to right. In the lowest slice an artifact occurred in the top‐right corner. All maps and images are in neurological display, as indicated by the “R” and “L” above the T_1_‐weighted images.

The mean CRLB values of total choline (tCho) and total creatine (tCr) (for the patterns that minimize the RMSE) are provided in Figure 3d. tNAA was not included, because the remaining lipid aliasing often superimposes the NAA peak, which could mistakenly decrease the CRLB values, thus making the tNAA CRLB inappropriate measures to determine the performance of PI methods. However, tNAA could be fitted properly, as shown in Figures 4, 5, 6, 7. The CRLB values of (2 + 1)D‐CAIPIRINHA were lower than those of 2D‐CAIPIRINHA only for R_Total_ ≥ 9, but they were very similar for the other acceleration factors.

Examples of metabolic maps of tNAA, tCr and tCho of two volunteers are shown in Figure 4 for the three PI methods and the fully sampled data set. Spectra are provided in Figure 5.

In summary, the mean AP, median g‐factor, absolute errors (same as RMSE without summing over the voxels), and CRLB values are provided in Supporting Table S1, together with the significance levels of the t‐test (AP, CRLB) or Wilcoxon signed rank tests (others). The four (AP and g‐factors) or five (RMSE and CRLB) volunteers and all of their voxels were considered the test samples, except for the APs, which inherently sums over all voxels. Because the voxel values are not entirely independent measurements, but the Wilcoxon and t‐tests require that condition, stronger significance‐level requirements were used.

### Lipid Contamination and Feasibility of (2 + 1)D‐CAIPIRINHA

Figure 6 shows an example of a tNAA map of Volunteer #5, which is strongly altered by lipid artifacts. The strong lipid artifacts, caused by a GRAPPA acceleration of R_Total_ = 10, were almost removed by the use of the lipid decontamination method.

The mean lipid and macromolecule values of all volunteers with and without lipid decontamination are given in Table 2 for all R_Total_s and all three acceleration methods.

**Table 2:** Lipid Contamination Ratio of R_Total_ = 1 for Different Acceleration Factors, Acceleration Methods, and with and without Lipid Decontamination, Averaged over Five Volunteers

	Lipid Ratio to R_Total_ = 1 [%]					
	Without lipid decontamination			With lipid decontamination		
	GRAPPA	CAIPI	(2 + 1)D‐C	GRAPPA	CAIPI	(2 + 1)D‐C
R = 2	98.9	101.0	—	60.2	59.8	—
R = 3	107.7	105.0	—	61.1	60.6	—
R = 4	109.1	113.4	—	62.0	61.8	—
R = 5	125.0	116.9	122.7	63.2	63.1	61.7
R = 6	136.7	126.6	131.7	65.1	65.0	58.6
R = 7	143.8	133.2	138.2	65.9	66.0	63.3
R = 8	151.5	160.0	149.7	67.3	67.1	72.1
R = 9	163.4	170.3	147.6	69.9	69.3	72.2
R = 10	192.0	198.2	180.4	71.9	72.0	77.6

Note: The lipid contamination increases with R_Total_, but is always well below the reference when using lipid decontamination. The lipid decontamination appears to work worse for (2 + 1)D‐CAIPIRINHA for R > 7, as the lipid values are higher than those of the other two methods, but lower when not using lipid decontamination.

In Figure 7, metabolic maps of tNAA, tCho, tCr, myo‐Inositol (Ins), and glutamine and glutamate (Glx) are shown for Volunteer #6, who was measured with actual (2 + 1)D‐CAIPIRINHA undersampling of R_Total_ = 8 and four slices within 15 min. The maps show a very good quality, with increases of tCho in the frontal and central brain area, consistent with other studies 54. The maps are ordered to increase in gray matter/white matter contrast from left to right, which is especially evident in Glx. The decreased metabolic concentrations in Slice 4 stem from a decreased sensitivity of the array coil at lower positions, which does not appear to be fully corrected during coil combination. The minor hotspots in the tNAA maps appear to be natural variations, as they also appear in another measurement of the same volunteer.

## DISCUSSION

In our work, we proposed a new PI method for multislice 2D‐^1^H‐MRSI that accelerates in all three spatial dimensions. Taking advantage of such acceleration results in maximal exploitation of the sensitivity variations of the AC, leading to expected reconstruction improvements over conventional PI methods. This theoretical expectation was confirmed by this study, as the proposed method provides lower AP, g‐factor, RMSE, and (partially) CRLB values, compared with 2D‐GRAPPA and 2D‐CAIPIRINHA. With the proposed method, accelerations up to R = 8 were feasible when using lipid decontamination according to Bilgic et al 53. Such high accelerations result in spectra with low SNR, allowing reliable fits (CRLBs < 20%) only for tCr, tCho, tNAA, Ins, and Glx (for the latter two, consider the ultrashort TE).

### Identifying the Best Undersampling Patterns

An analysis of Step 2 showed that the APs followed the expected trend almost perfectly: The higher the R_Total_, the higher the AP for all three PI methods. Moreover, (2 + 1)D‐CAIPIRINHA led to lower APs than 2D‐CAIPIRINHA, as it makes better use of the AC in all three dimensions. However, substantial differences in AP between 2D‐CAIPIRINHA and (2 + 1)D‐CAIPIRINHA were found only for high R_Total_ values, because, only for high acceleration were the in‐plane sensitivity variations no longer sufficient for 2D‐CAIPIRINHA using our 32‐channel receive coil. 2D‐CAIPIRINHA provided lower APs than 2D‐GRAPPA. This is in accordance with the literature 23.

The g‐factors showed a similar behavior, with the exception of R_Total_ = 9, in which all methods performed equally well. Moreover, the g‐factor decrease of the 2D‐GRAPPA method from R_Total_ = 7 to R_Total_ = 9 is worth noting. These patterns were achieved with the same pattern as for R_Total_ = 9, ie, a 3 × 3 pattern, but with higher VD radii. It is known that the best SNR performance is achieved by sampling the k‐space uniformly. With high VD radii, this is not the case, thus, resulting in lower SNR/t and higher g‐factors. The high g‐factor increase between R_Total_ = 9 and R_Total_ = 10 was caused by the high acceleration factor of 4 in one direction of the 4 × 3 or 3 × 4 patterns.

### Analyzing the Best Undersampling Patterns

Step 3 showed that (2 + 1)D‐CAIPIRINHA provides overall better RMSE values. The CRLB values were similar for 2D‐CAIPIRINHA and (2 + 1)D‐CAIPIRINHA for R_Total_ < 9, but better than for 2D‐GRAPPA. Only at very high R_Total_ (>7) was (2 + 1)D‐CAIPIRINHA better than the other two PI methods. The qualitative assessment of metabolic maps and spectra showed very similar results for 2D‐CAIPIRINHA and (2 + 1)D‐CAIPIRINHA for a low R_Total_, but better performance for R_Total_ > 7. The performance versus 2D‐GRAPPA was better for all R_Total_ (>4).

2D‐CAIPIRINHA also performed better than 2D‐GRAPPA. This can be attributed to the fact that 2D‐CAIPIRINHA can achieve a more even distribution of the aliasing across the whole FOV than 2D‐GRAPPA.

### Lipid Contamination and Feasibility of (2 + 1)D‐CAIPIRINHA

The lipid decontamination provided good results, decreasing the mean lipid and macromolecule contents from almost 200% to under 80% of that of R_Total_ = 1. Note that the lipid and macromolecule ratio of 200%, on average, has to be considered a substantial increase, as most voxels are usually not affected at all, whereas some regions are likely to be highly contaminated and may thus be unusable. It is further important to stress that 80% can be considered a low value, because even for perfect lipid suppression, the choice to sum the signal between 0.3 and 2.1 ppm overestimates the lipid signal. Furthermore, with 80% the lipid contamination is lower than for R = 1 without lipid suppression, in which case lipids are no problem because of the high resolution. Despite GRAPPA working well in general, the remaining lipid artifacts are especially problematic with GRAPPA at high acceleration factors if no lipid suppression method is used.

The successful measurement in a volunteer acquired with (2 + 1)D‐CAIPIRINHA provided proof‐of‐principle of the proposed method. Even Ins and Glx were adequately fittable in all slices, showing a strong gray and white matter contrast. Using slice gaps of 50% was not superior to using no slice gap in a prestudy 55; therefore, no slice gap was used in the measurement.

### Comparison of PI Methods

To date, two methods to accelerate in all three spatial dimensions have been proposed in conventional MRI: Zigzag sampling by Breuer et al 45 and Wave‐CAIPI by Bilgic et al 44. Both involve shifting the individual frequency‐encoding k‐space points along the phase‐encoding directions by applying time‐varying phase‐encoding gradients during the readout.

In conventional MRSI, it is possible to accelerate in three dimensions without any additional shifts. To date, at most, two spatial dimensions have been exploited for PI acceleration in conventional MRSI 35, 38, 42, 56, using only standard GRAPPA 14, 29, 40, 41, 42 and SENSE 28, 29, 30, 31, 32, 33, 34, 35, 36, 37, 38, 39. SENSE, in its normal, so‐called “strong” approach, was shown to be prone to reconstruction errors when applied to low‐resolution data 34. Some of the groups who performed GRAPPA measured the ACS data with a time‐consuming MRSI sequence 29, 41, 42, although there is no evidence that calculating GRAPPA weights for each spectral time point improves the reconstruction.

In contrast, our MRSI data were reconstructed using a k‐space‐based reconstruction. In this way, we avoided the point‐spread function complications of the SENSE reconstruction 34. In addition, the ACS data were acquired with an imaging‐based sequence, which requires only a few seconds. No lengthy, inefficient MRSI measurements of the central k‐space were required for the ACS data. These were acquired with a sequence very similar to that of the MRSI data, and with a high matrix size of 128 × 128, in contrast to matrix sizes of approximately 32 × 256 in MRI. Both facts are beneficial for the PI reconstruction. Through PI acceleration in all three spatial dimensions, including 2D‐CAIPIRINHA patterns superior to standard GRAPPA patterns, we avoided additional SNR losses as a result of increased g‐factors. This is particularly important if high acceleration factors are used. Acceleration factors up to 8 (Volunteer #6) or 10 (simulations) were therefore possible in vivo, because of the high SNR provided by the ultrahigh magnetic field, the AC with 32 channels, the optimal coil combination 8, and the ultrashort acquisition delay 51.

Because of the FOV shifts between the aliased slices, the sensitivity variation is exploited not only along the slice direction, but also along the PE directions in the 1D‐CAIPIRINHA reconstruction, thus requiring no slice gaps. Large distances between the aliased slices are, however, beneficial. The proposed method is therefore best suited for measurements with big slice gaps, 2–8 slices, and high acceleration factors.

One additional feature that was used in our work was Hadamard encoding of the different aliased slice groups. In Figure 1a, this would be the group consisting of Slices 1 and 3, and the group consisting of Slices 2 and 4. Slice‐CAIPIRINHA can be combined with Hadamard encoding, because slice‐CAIPIRINHA adds a linearly increasing phase to the excitation pulse for the different phase‐encoding points. Hadamard encoding, however, adds a constant phase to the excitation pulse phase for all phase‐encoding points, but different phases for different Hadamard steps.

All GRAPPA‐like reconstruction methods, and therefore all three methods compared here, could be further improved by a regularized PI reconstruction 57, or by calculating different GRAPPA weights for different regions of the k‐space 58.

### Comparison to SSE in MRSI

SSE is a good alternative to PI for accelerating MRSI. SSE techniques can provide higher acceleration factors than PI, and do not suffer from lipid aliasing, while still maintaining an SNR/t close to that of conventional MRSI. A minor disadvantage of SSE is the high load on the gradient coils, which can cause frequency drifts and associated line broadening at 3 T 59, 60. These effects are further worsened at 7 T because of the shortened spectral dwell times required to cover the same spectral range in parts per million.

### Limitations

The main limitations of this study are the possible lipid contaminations that occur when the acquired ACS data and MRSI data differ as a result of subject motion and other instabilities, and the longer reconstruction times compared with 2D‐GRAPPA. A large volume was carefully shimmed to prevent the subcutaneous lipids from resonating within the metabolite chemical shift range and a lipid decontamination was used in postprocessing, based on an L2‐norm‐regularized reconstruction proposed by Bilgic et al 53. Other solutions will be considered in future studies, such as dedicated gradient crusher coils 61, higher order shims 62, constrained shimming routines 26, outer volume saturation 63, and (double) inversion recovery methods 64, 65.

The proposed method is only reasonable for eight slices or fewer, because pulse‐cascaded Hadamard introduces acquisition delays that become longer with the number of slices, and the slice‐acceleration is limited to approximately R_Slc_ = 2. For more slices, 3D‐CAIPIRINHA would be the better choice. This is a distinct disadvantage compared with SSE methods, which can easily provide accelerations higher than 10, and thus more slices within a reasonable time.

It is crucial to understand that the optimal patterns found in our study are not necessarily optimal if other coils or slice positions are used, or if investigating different organs. In particular, if the coil arrangement along the *z*‐dimension differs substantially, the optimal patterns will be different. Lower field strengths should theoretically not influence the choice of the best patterns, but all patterns should provide worse reconstructions as a result of more overlapping sensitivities. The best patterns must be identified for each coil and organ individually, which is in fact also the case for 2D‐CAIPIRINHA and, to a small extent, even for 2D‐GRAPPA (2 × 3 versus 3 × 2). However, simulations based on the Biot‐Savart law can be performed to restrict the number of patterns to begin with. The exact patterns are provided as Supporting Material 2, because for coil geometries similar to ours and for brain measurements, the patterns found in this study are likely to be among the best choices.

The long reconstruction times of all three compared methods could be mitigated by improving the reconstruction algorithm.

## CONCLUSIONS

In this study, we show, for the first time, the feasibility of PI along three spatial dimensions in MRSI. (2 + 1)‐CAIPIRINHA can be implemented for multislice MRSI in the brain, enabling higher accelerations than are possible with 2D parallel imaging methods. (2 + 1)D‐CAIPIRINHA is therefore the preferred method, if high acceleration factors are required for multislice MRSI.

## Supporting information

Additional Supporting Information may be found in the online version of this article

**Table S1.** Mean APs, Median g‐Factors, Median Absolute Errors, and Mean CRLBs of tCho and tCr for the Three Methods and Six Acceleration Factors (Bold numbers indicate that the reference method was worse than (2 + 1)D‐CAIPIRINHA; * indicates statistical significance (*P* < 5·10^−2^ for AP, *P* < 5·10^−3^ for the others); and ** indicates highly significant differences (*P* < 5·10^−4^). A t‐test was performed on the AP data of the five volunteers. For the other quality measures, the voxels of all volunteers were used as the sample for the t‐test (CRLB values) or Wilcoxon signed rank test (g‐factors, median absolute errors), which requires independent measurements. Because the voxel values are not entirely independent (only the k‐space data are), stronger significance level requirements were chosen.)

**Supporting Material 2.** MATLAB file containing the best 2D‐GRAPPA, 2D‐CAIPIRINHA, and (2 + 1)D‐CAIPIRINHA Patterns after Step 3. In the latter case, a 2D‐CAIPIRINHA pattern and two FOV shifts are provided. The FOV shifts are given as multiples of the FOV (eg, a value of 0.5 indicates half a FOV shift). The 2D‐CAIPIRINHA patterns consist of 0s and 1s, in which 0s represent not measured, and 1s represent measured k‐space points. These patterns have to be replicated to the intended matrix size (eg, from 5 × 5 to 64 × 64). The VD radius gives the radius in multiples of 1/FOV, within which all k‐space points are measured.
