# Supplementary material for: (2 + 1)D‐CAIPIRINHA accelerated MR spectroscopic imaging of the brain at 7T
Source: Magn Reson Med. 2016 Aug 22;78(2):429–40. doi: 10.1002/mrm.26386 (PMC5535010; doi:10.1002/mrm.26386)
Supplement: Supplementary file 1 — Table S1. Mean APs, Median g‐Factors, Median Absolute Errors, and Mean CRLBs of tCho and tCr for the Three Methods and Six Acceleration Factors (Bold numbers indicate that the reference method was worse than (2 + 1)D‐CAIPIRINHA; * indicates statistical significance (P < 5·10−2 for AP, P < 5·10−3 for the others); and ** indicates highly significant differences (P < 5·10−4). A t‐test was performed on the AP data of the five volunteers. For the other quality measures, the voxels of all volunteers were used as the sample for the t‐test (CRLB values) or Wilcoxon signed rank test (g‐factors, median absolute errors), which requires independent measurements. Because the voxel values are not entirely independent (only the k‐space data are), stronger significance level requirements were chosen.) [file MRM-78-429-s001.doc]

|  |  | R=5 | R=6 | R=7 | R=8 | R=9 | R=10 |
| --- | --- | --- | --- | --- | --- | --- | --- |
| AP [%] | GRAPPA | **8.86*** | **9.89** | **12.54*** | **13.22*** | **14.18** | **17.94** |
|  | CAIPI | **6.97** | 8.42 | **9.86** | **11.56** | **13.51** | **15.14*** |
|  | (2+1)-CAIPI | 6.68 | 8.47 | 9.63 | 10.31 | 11.13 | 13.06 |
| g-Factor [] | GRAPPA | 1.10** | **1.10**** | **1.18**** | 1.16** | 1.14** | **1.30**** |
|  | CAIPI | 1.08** | **1.11**** | 1.12** | 1.12** | 1.14 | **1.21**** |
|  | (2+1)-CAIPI | 1.10 | 1.08 | 1.12 | 1.17 | 1.14 | 1.15 |
| Absolute Error [%] | GRAPPA | **6.0**** | **7.3**** | **8.2**** | **8.8**** | **9.5**** | **11.7**** |
|  | CAIPI | 5.7 | **6.9**** | **8.0**** | **8.7**** | **9.5**** | **11.3**** |
|  | (2+1)-CAIPI | 5.9 | 6.5 | 7.2 | 8.4 | 8.5 | 10.0 |
| CRLB [%] | GRAPPA | 6.37 | **6.71** | **7.35**** | **7.57** | **7.78**** | **8.85**** |
|  | CAIPI | 6.18** | 6.58 | 7.01 | 7.30** | **7.91**** | **8.57**** |
|  | (2+1)-CAIPI | 6.37 | 6.66 | 7.10 | 7.50 | 7.57 | 8.21 |

Supporting Table S1: The mean APs, median g-factors, median absolute errors, and mean CRLBs of tCho and tCr for the three methods and six acceleration factors. Bold numbers indicate that the reference method was worse than (2+1)D-CAIPIRINHA, * indicates statistical significance (p < 5**.**10^-2^ for AP, p < 5**.**10^-3^ for the others), and ** indicates highly significant differences (p < 5**.**10^-4^). A t-test was performed on the AP data of the five volunteers. For the other quality measures, the voxels of all volunteers were used as the sample for the t-test (CRLB values) or Wilcoxon signed rank test (g-factors, median absolute errors), which requires independent measurements. Since the voxel values are not entirely independent (only the k-space data are), stronger significance level requirements were chosen.
